# Prolonged T_peak_‐T_end_ interval is a risk factor for sudden cardiac death in adults with congenital heart disease

**DOI:** 10.1111/chd.12847

**Published:** 2019-10-01

**Authors:** Jim T. Vehmeijer, Zeliha Koyak, A. Suzanne Vink, Werner Budts, Louise Harris, Candice K. Silversides, Erwin N. Oechslin, Aeilko H. Zwinderman, Barbara J.M. Mulder, Joris R. de Groot

**Affiliations:** ^1^ Department of Clinical and Experimental Cardiology Heart Center Amsterdam University Medical Centers University of Amsterdam Amsterdam the Netherlands; ^2^ Department of Cardiology Universitair Ziekenhuis Leuven Leuven Belgium; ^3^ Department of Cardiovascular Sciences Katholieke Universiteit Leuven Leuven Belgium; ^4^ Division of Cardiology Peter Munk Cardiac Centre Toronto Congenital Cardiac Centre for Adults University of Toronto Toronto Ontario Canada; ^5^ Department of Clinical Epidemiology and Biostatistics Amsterdam University Medical Centers University of Amsterdam Amsterdam the Netherlands; ^6^ Netherlands Heart Institute Utrecht the Netherlands

**Keywords:** congenital heart disease, electrocardiography, risk factor, sudden cardiac death, Tpeak‐Tend

## Abstract

**Objective:**

Adult congenital heart disease (ACHD) patients are at risk of sudden cardiac death (SCD). However, methods for risk stratification are not yet well‐defined. The T_peak_‐T_end_ (TpTe) interval, a measure of dispersion of ventricular repolarization, is a risk factor for SCD in non‐ACHD patients. We aim to evaluate whether TpTe can be used in risk stratification for SCD in ACHD patients.

**Design:**

From an international multicenter cohort of 25 790 ACHD patients, we identified all SCD cases. Cases were matched to controls by age, gender, congenital defect, and (surgical) intervention.

**Outcome Measures:**

TpTe was measured on a standard 12‐lead ECG. The maximum TpTe of all ECG leads (TpTe‐max), mean (TpTe‐mean), and TpTe dispersion (maximum minus minimum) were obtained. Odds ratios (OR) for SCD cases vs controls were calculated using conditional logistic regression analysis.

**Results:**

ECGs were available for 147 cases (median age at death 33.5 years (quartiles 26.2, 48.7), 66% male) and 267 controls. The mean TpTe‐max was 97 ± 24 ms in cases vs 84 ± 17 ms in controls (*P* < .001); TpTe‐mean was 70 ± 16 vs 63 ± 10 ms (*P *< .001); and dispersion was 51 ± 22 ms vs 41 ± 16 ms (*P* = .02), respectively. Assessing each ECG lead separately, TpTe in lead aVR predicted SCD most accurately. TpTe in lead aVR was 71 ± 23 ms in cases vs 61 ± 13 ms in controls (*P* < .001). After adjusting for impaired ventricular function, heart failure symptoms, and prolonged QRS duration, the OR of SCD of TpTe in lead aVR at an optimal cutoff of 80 ms was 5.8 (95% CI 2.7‐12.4, *P* < .001).

**Conclusions:**

The TpTe interval is associated with SCD in ACHD patients. Particularly, TpTe in lead aVR can be used as an independent risk factor for SCD in ACHD patients and may, therefore, add precision to current risk prediction models.

## 1 INTRODUCTION

Adult congenital heart disease (ACHD) patients are at risk of a myriad of complications and causes of death.^1^, ^2^, ^3^, ^4^ One of the leading causes of mortality in these patients is sudden cardiac death (SCD),^5^ which accounts for 20%‐25% of all deaths in ACHD patients. Conversely, congenital heart disease is found to be the underlying disease in 22% of all SCD cases in 18‐to 25‐year olds and 13% of 26‐to 34‐year olds.^3^, ^5^, ^6^ SCD in ACHD patients is mainly caused by ventricular arrhythmias and, thus, may be very effectively prevented by implanting an implantable cardioverter‐defibrillator (ICD).^5^, ^7^, ^8^ However, accurate prediction of SCD in ACHD patients remains difficult due to the relative rarity of the event. It is, therefore, challenging to predict who will benefit from ICD therapy. Efforts have been made in the past years to identify ACHD patients at increased risk of SCD and to give guidance in the management of these patients. Three guideline documents have recently listed recommendations for primary prevention ICD implantation for ACHD patients specifically.^9^, ^10^, ^11^ In these documents, risk stratification is based on established risk factors for acquired heart disease and includes impaired systemic ventricular function, heart failure symptoms, and prolonged QRS duration. These risk factors seem to be of importance in ACHD patients as well.^5^, ^12^, ^13^, ^14^ However, when applied in a case‐control cohort, the current guideline recommendations for ICD implantation showed a poor ability to discriminate SCD cases from controls.^15^ Thus, the risk factors used in acquired heart disease may not suffice to accurately identify high‐risk ACHD patients who may get benefit from ICD therapy. Therefore, it is imperative to optimize the risk stratification in ACHD patients and determine new risk factors.

In a previous study, several electrocardiographic parameters were measured, including the QT interval, and compared between SCD cases and living controls. The QT interval did not differ significantly between cases and controls.^5^ The T_peak_‐T_end_ (TpTe) interval may provide a more accurate measure of dispersion of ventricular repolarization and is a risk factor for ventricular arrhythmia and SCD in patients with genetic arrhythmia syndromes as well as those with acquired heart disease.^16^, ^17^, ^18^, ^19^, ^20^, ^21^, ^22^ It is also associated with appropriate ICD shocks in ACHD patients.^23^ It may, therefore, also be a predictor for SCD in ACHD patients. In this study, we aimed to assess the predictive ability of TpTe for SCD in a cohort of ACHD patients who died of SCD and living controls.

## 2 METHODS

This retrospective case‐control study used a multicenter, international cohort of ACHD patients (≥18 years old). The cohort included patients from the Dutch CONCOR registry, Toronto Congenital Cardiac Centre for Adults, and the University Hospitals Leuven, and comprised a total of 25 790 ACHD patients. As part of a previous study, all patients with tachyarrhythmic SCD (*n* = 171) were included.^5^ SCD was defined as (1) proven or documented arrhythmic death, (2) arrhythmic death by exclusion (instantaneous death or circumstances compatible with SCD, without disease that would lead to death in the near future, and the absence of a non‐arrhythmic cause of death at autopsy), or (3) arrhythmic death by default: abrupt loss of consciousness and absence of pulse, without further data.

SCD cases were matched to controls at a 1:n ratio (up to three controls per case, depending on availability) by (1) age (+ or − 5 years of corresponding SCD case), (2) gender, (3) congenital defect, (4) type of surgical intervention (eg, prior shunt, palliative or corrective surgery, valve replacements), (5) date of surgical repair (+ or − 5 years), and (6) treating medical center. The study design and population have been presented elsewhere in detail.^5^, ^24^ In this study, we included all cases with an ECG and their respective controls.

### 2.1 Data collection and TpTe measurement

We obtained approval from the medical ethics committees in the participating centers. In the included SCD cases, we acquired the last standard 12‐lead ECGs before death. In controls, we included the ECG included that was made at the age closest to their respective cases’ age at the last ECG before death.

A physician with experience in ECG analysis (J.V.), blinded for clinical characteristics and outcomes of patients, measured TpTe using the Fiji distribution of ImageJ, a widely used biomedical image analysis tool designed by the U.S. National Institutes of Health.^25^ The investigator measured TpTe in one T‐wave of each of the 12 leads of the standard ECG. We defined TpTe as the interval between the peak of the T‐wave (T_peak_) and the end of the T‐wave (T_end_). We defined T_peak_ as the point in the T‐wave the farthest from the baseline, either in a positive or negative deflection. We determined T_end_ using the tangent method.^26^ Out of the total of 12 measurements on each ECG, we determined the maximum (TpTe‐max) and minimum TpTe (TpTe‐min) and calculated the mean TpTe (TpTe‐mean) and TpTe dispersion (maximum minus minimum). Additionally, we attempted to find the optimal cutoff for a binary variable of TpTe by assessing where the OR for SCD of patients with TpTe above the cutoff value was the highest, at 10 ms increments.

In order to obtain interobserver validity, a second, independent, and blinded observer (ASV) assessed and analyzed a random sample of 43 ECGs (over 500 measurements).

### 2.2 Statistical analysis

We performed all data analyses using R (version 3.5.0, R Foundation for Statistical Computing, Vienna, Austria) and SPSS (IBM SPSS Statistics for Windows, Version 24.0. IBM Corp. Armonk, NY).^27^ We expressed descriptive statistics for nominal data as absolute numbers and percentages. For normally distributed continuous variables, we calculated mean values and standard deviations (SDs). We presented non‐normally distributed data in medians and interquartile range (IQR). In order to correct for the difference in number of controls per case, we used conditional logistic regression models for the analysis of differences in patient characteristics and for analysis of risk factors for SCD and displayed the results in odds ratios (OR) with 95% confidence intervals (CI). To adjust the TpTe for other risk factors for SCD, that is, impaired systolic systemic ventricular function, defined as a systemic ventricular ejection fraction <39%, the presence of heart failure symptoms and a prolonged QRS complex of >120 ms, we performed multivariable regression analyses. Subsequently, we used the likelihood ratio test to calculate *P* values associated with these models. To find the additional value of TpTe in a multivariable model with the aforementioned other risk factors, we assessed the area under the receiver operator characteristic curve (C‐statistic). Because TpTe is a manual measurement, we assessed the interobserver validity with the intraclass correlation coefficient (ICC) for multiple measurements based on a two‐way agreement model. We used conditional logistic regression with interaction terms to establish whether the RR, QRS, or QT interval or ventricular pacing had a significant interaction with TpTe for the outcome of SCD. For all analyses, we considered two‐tailed *P* values < .05 to be statistically significant.

## 3 RESULTS

### 3.1 Baseline characteristics

**Figure 1:**
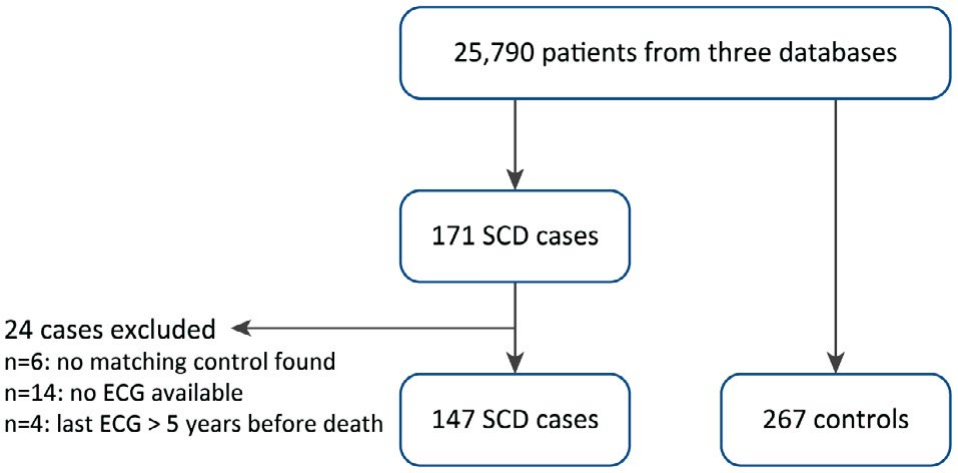
Flowchart of patient selection. Patients are included from the Dutch CONCOR registry, Toronto Congenital Cardiac Centre for Adults, and the University Hospitals Leuven

**Figure 2:**
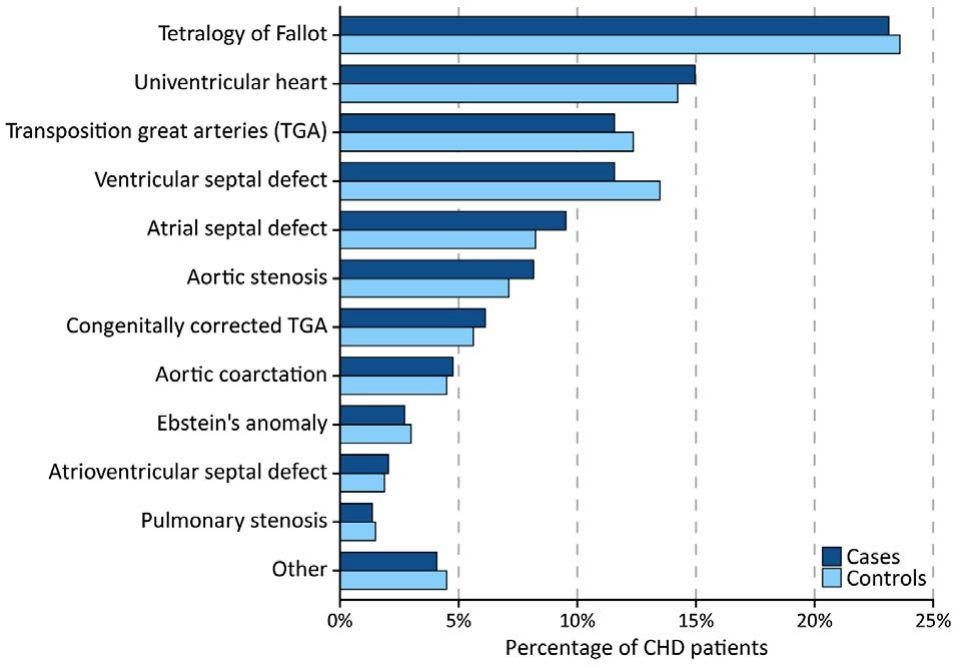
Proportions (%) of different defects in cases and controls

Of the 171 SCD cases in the total cohort, an ECG was available in 147 cases. As a consequence, 267 respective controls could be included. A flowchart of patient selection is presented in Figure 1. Sixty‐five percent of SCD cases were male, and the median age was 33.5 years (IQR 26.2‐48.7). Other characteristics of cases and controls are presented in Table 1, and the underlying congenital heart defects are shown in Figure 2.

**Table 1:** Characteristics of cases and controls

	Case	Control	*P* value
*n*	147	267	
Age, median (IQR)	34 (26, 49)	34 (27, 46)	.957
Female, *n* (%)	51 (35)	96 (36)	.831
Impaired SVF, *n* (%)	52 (35)	33 (12)	<.001
Heart failure symptoms, *n* (%)	84 (57)	85 (32)	<.001
QRS > 120 ms, *n* (%)	89 (61)	111 (42)	<.001

Abbreviations: IQR, interquartile range; SVF, systolic systemic ventricular function.

### 3.2 TpTe interval

Of the total 414 included ECGs, the TpTe interval could be determined in all 12 leads in 389 ECG (94%, 139 cases and 250 controls). TpTe could not be observed in some leads in the remaining ECGs due to flat T‐waves or distortion from atrial flutter waves. There was a good interobserver validity with an ICC of 0.70 (95% CI 0.52‐0.79). There was no significant interaction of the RR, QRS, or QT interval with TpTe for the outcome of SCD. Finally, because ventricular pacing may also influence TpTe, we checked for interaction between a ventricular paced rhythm and TpTe. Seventeen percent of cases and thirteen percent of controls had a ventricular paced rhythm, but there was no significant interaction between TpTe and a ventricular paced rhythm (interaction *P* = .85).

**Figure 3:**
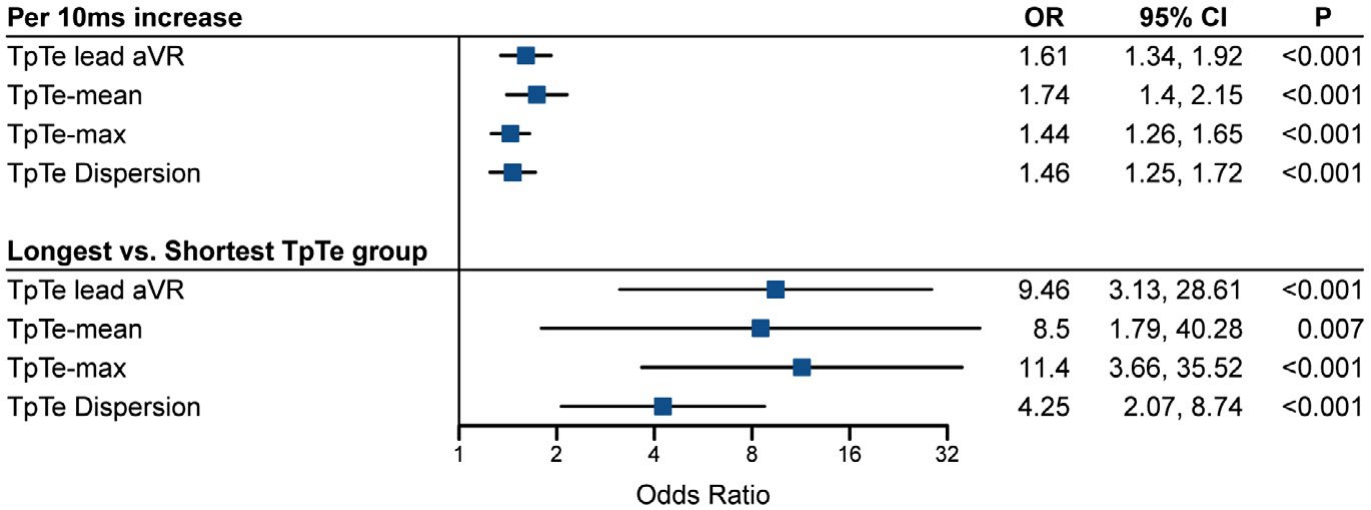
Forest plot of univariable ORs for SCD of TpTe measurements grouped per 10 ms increase. Notres: TpTe‐mean and TpTe lead aVR: < 50, 50‐59, 60‐69, 70‐79, 80‐89, and ≥90 ms, TpTe‐max: <70, 70‐79, 80‐89, 90‐99, 100‐109, 110‐119, and ≥120 ms, TpTe dispersion: <30, 30‐39, 40‐49, 50‐59, and ≥60 ms

The mean TpTe‐max was significantly longer in cases (97 ± 25) than in controls (84 ± 17 ms), *P* < .001, as was TpTe‐min: 46 ± 13 ms vs 43 ± 10 ms, respectively, *P* = .02. TpTe‐mean was also longer in cases than in controls: 70 ± 16 ms versus 63 ± 10 ms, respectively, P < .001, and cases had a greater TpTe dispersion: 51 ± 21 vs 41 ± 16 ms, respectively (*P* < .001). The ECG lead in which the TpTe interval was most strongly associated with SCD was lead aVR (TpTe‐aVR) (Supplemental Figure S1). In addition, TpTe measurement in lead aVR had a very good interobserver validity with an ICC of 0.82 (95% CI 0.60‐0.91). The OR of SCD per 10 ms for TpTe‐max, TpTe‐mean, TpTe dispersion, and TpTe in aVR are shown in the upper panel of Figure 3.

In the multivariable analysis, adjusted for systemic ventricular dysfunction, heart failure, and QRS duration >120 ms, the OR of SCD of TpTe‐max was per 10 ms increase was 1.42 (95% CI 1.2‐1.7, *P* < .001). Likewise, the OR of SCD of TpTe‐min was 0.99 (95% CI 0.7‐1.4, *P* = .9), for TpTe‐mean was 1.6 (95% CI 1.2‐2.0, *P* < .001), and for TpTe dispersion was 1.49 (95% CI 1.2‐1.8, *P* < .001).

In addition, we tested the optimum cutoff point for a binary variable of TpTe‐mean. In univariable analysis, the higher the cutoff was set, the greater the OR of SCD was up to a cutoff of 90 ms. A total of 10 cases, but none of the controls had a TpTe‐mean >100 ms. In multivariable analysis, a cutoff of TpTe‐mean of 70 ms had the highest OR of SCD (Supplemental Figure S2).

The multivariable adjusted OR of SCD of TpTe‐aVR ≥80 ms vs <80 ms was 5.8 (95% CI 2.7‐12.4, *P* < .001). Although a cutoff point of 90 ms reached a higher OR (adjusted OR 11.3), only 18% of cases and 3% of controls fell above this range, compared to 31% and 8%, respectively, at a cutoff of 80 ms (Supplemental figure S3). In a multivariable analysis of a model consisting of impaired ventricular function, heart failure symptoms, and QRS duration >120 ms, the addition of TpTe in aVR >80 ms increased the C‐statistic of the model from 0.696 (95% CI 0.642‐0.750) to 0.742 (95% CI 0.690‐0.794).

### 3.3 TpTe measurement in subgroups

In a subset of patients with an impaired systolic systemic ventricular function, the OR for SCD of TpTe‐mean was 1.7 (95% CI 0.88‐3.1, *P* = .075) per 10 ms increase. TpTe‐aVR had an OR for SCD of 2.1 (95% CI 0.98‐4.6, *P* = .023) per 10 ms increase.

In the largest group of patients with the same congenital defect, tetralogy of Fallot (TOF, *n* = 34 SCD cases), the OR for SCD of TpTe‐mean per 10 ms increase was 1.5 (1.0‐2.3, *P* = .03), and for TpTe‐aVR was 1.8 (1.2‐2.6, *P* < .001). In univentricular hearts (UVH, *n* = 22 cases), this OR was 1.8 (1.1‐3.0, *P* = .02) and 1.5 (0.96‐2.3, *P* = .06), respectively. In transposition of the great arteries (TGA, *n* = 17 cases), the OR of SCD of TpTe‐mean was 1.9 (1.0‐3.5, *P* = .02) and of TpTe‐aVR was 1.5 (0.9‐2.63, *P *= .125) per 10 ms increase.

## 4 DISCUSSION

### 4.1 Main findings

This study shows that a prolonged TpTe interval is associated with SCD in a cohort of ACHD patients, and that especially a TpTe in lead aVR ≥80 ms was a strong predictor. TpTe measurement in lead aVR is a simple, non‐invasive measurement with a very good interobserver validity. It can be measured on a standard 12‐lead ECG, which is usually readily available in all ACHD patients. Therefore, it may serve as a convenient risk stratification tool for SCD in ACHD patients in daily practice. The TpTe interval showed an incremental risk of SCD per 10 ms increase. Consequently, when compared to patients with the shortest TpTe, the patients with the longest TpTe had a substantially increased risk of SCD (OR of TpTe‐aVR ≥90 ms vs <50 ms 7.9, Figure 3).

We have shown that the TpTe in lead aVR is the most viable parameter for risk stratification, since it requires assessment of only one ECG lead, and performed better than the TpTe‐mean in most cases. A reason for this may be that lead aVR is a unique lead on the ECG: other ECG leads provide information on specific parts of the myocardium, whereas lead aVR gives a more general overview of the heart. In patients with global ischemia, for example, left main coronary artery occlusion, only lead aVR shows ST‐elevation, whereas other leads show ST‐depression.^28^ Lead aVR may also be of a better quality and have a greater stability of the electrical signal than other ECG leads, making TpTe measurement in lead aVR more accurate.

### 4.2 TpTe as a predictor of SCD

TpTe prolongation is a sign of dispersion of repolarization. Previous studies have shown that it may either demonstrate transmural dispersion of repolarization due to prolonged repolarization of the subendocardial M‐cells, or overall dispersion of repolarization.^17^, ^20^, ^29^ As reentry requires unidirectional block and slow conduction, increased dispersion of refractoriness increases the vulnerability of the myocardium for reentrant tachycardias, because the conditions under which these may occur are present during a longer time.

When adjusting TpTe for the established risk factors, that is, impaired ventricular function, heart failure symptoms, and prolonged QRS duration, TpTe remained strongly associated with SCD. TpTe may, therefore, add to currently existing risk score models, which usually consist of these parameters.^9^ This is of importance, since the currently existing models are certainly in need of improvement, since they only correctly identify about 35%‐40% of SCD cases.^15^ Adding TpTe to a multivariable model consisting of these risk factors produces a meaningful increase of the C‐statistic.

ACHD is characterized by a high variability in anatomy, hemodynamics, surgical repair, and medical treatment. It is, therefore, not surprising that no single risk factor has thus far been described that accurately predicts SCD in these patients. The use of prediction models or schemes of several individual risk factors may help identify these patients in whom preventive measures against SCD are necessary. TpTe may be a robust addition to such risk models.

### 4.3 Comparison with other studies

Several studies have shown a correlation between a prolonged TpTe interval and SCD in patients with genetic arrhythmia syndromes and acquired heart disease^16^, ^18^, ^21^, ^30^. In these studies, a TpTe of >100 ms appeared to be an important risk factor for SCD. Indeed, when the cutoff point for a binary parameter of TpTe was set at 100 ms in our study, ten cases and zero controls fell above this threshold. In this study, cases had a mean TpTe‐mean of 70 ms. In multivariable analysis, cutoff of TpTe‐mean of 70 ms and TpTe‐aVR of 80 ms was the most predictive of SCD. A reason for this difference may be that there are two methods for manual assessment of the end of the T‐wave: the tangent and the threshold method.^26^, ^31^ We used the tangent method in this study; some other studies used the threshold method,^23^ while other studies did not define the method of measurement that was used for determination of the end of the T‐wave. It is, however, known that the tangent method consistently measures significantly shorter QT intervals than the threshold method, which is due to the differences in the determination of the end of the T‐wave.^31^

### 4.4 Strengths and limitations

The present study comprises the largest cohort of SCD ACHD patients thus far. It may, therefore, provide the most accurate results currently possible. However, congenital heart diseases are a highly heterogeneous patient group and it may not be possible to assess risk factors for SCD for each congenital defect independently. Contrary to other cohorts, that also measured death‐like events, such as appropriate ICD shocks and aborted cardiac arrest, this cohort only consists of patients who died of SCD. It may, therefore, provide more accurate data on the risk of death.

As we performed a retrospective case‐control study, it has the inherent limitation of a retrospective study, including risk of bias and its statistical limitations. Then, in some cases there was neither documentation of heart rhythm available, nor was autopsy performed in all cases. Therefore, some cases might not have died of tachyarrhythmic SCD. However, we did use the most common definition of SCD in this study.

The intraclass correlation of TpTe measurement was 0.7, signifying a good, but not optimal agreement between observers, although the intraclass correlation of TpTe in lead aVR was 0.82. Thus, TpTe measurement performed by a physician experienced in measuring congenital heart disease patients’ ECGs will likely be most accurate. On some ECGs, TpTe measurements were hampered by biphasic or flat T‐waves, which likely reduces the accuracy of TpTe. In addition, TpTe was usually not measurable in patients not in sinus rhythm.

Lastly, we cannot be certain that the results presented here can be extrapolated to all congenital heart disease patients. Because patients with simple defects have a lower risk of SCD, they are underrepresented in this cohort. Additionally, very rare congenital defects are underrepresented as well. However, since most high‐risk patients are included in this cohort, the effect of this underrepresentation on daily practice is likely to be minimal.

## 5 CONCLUSION

A prolonged TpTe interval is independently associated with SCD in ACHD patients. The independent OR of SCD of TpTe in lead aVR of ≥80 ms is 5.8. TpTe is a convenient, non‐invasive and accurate predictor with a good interobserver validity. In addition, since TpTe is an independent risk factor, it may add more precision to currently existing risk prediction models. Although TpTe may not be a strong enough predictor to warrant ICD implantation by itself, it may aid in the decision for ICD implantation.

## CONFLICT OF INTERESTS

J.T. Vehmeijer, Z. Koyak, A.S. Vink, L. Harris, C.K. Silversides, E.N. Oechslin, W. Budts, A.H. Zwinderman, and B.J. Mulder declare no conflicts of interest. J.R. de Groot receives unrestricted research grants from Medtronic, Abbott Laboratories, and Atricure and is a consultant at Daiichi Sankyo and Atricure.

## AUTHOR CONTRIBUTIONS

*Concept/Design*: J.T. Vehmeijer, J.R. de Groot

*Data analysis/interpretation*: J.T. Vehmeijer, A.S. Vink, A.H. Zwinderman

*Drafting article*: J.T. Vehmeijer

*Critical revision of article*: J.T. Vehmeijer, Z. Koyak, A.S. Vink, W. Budts, L. Harris, C.K. Silversides, E.N. Oechslin, A.H. Zwinderman, B.J.M. Mulder, J.R. de Groot

*Approval of article:* J.T. Vehmeijer, Z. Koyak, A.S. Vink, W. Budts, L. Harris, C.K. Silversides, E.N. Oechslin, A.H. Zwinderman, B.J.M. Mulder, J.R. de Groot

*Statistics:* J.T. Vehmeijer, A.S. Vink, A.H. Zwinderman

*Funding secured by:* J.R. de Groot; *Data collection:* J.T. Vehmeijer, A.S. Vink

## Funding information

This was an investigator‐initiated study. No specific funding was received for this study, other than from Academic Medical Center Medical Research BV. J.R. de Groot is supported by a Vidi grant from The Netherlands Organisation for Health Research and Development (ZonMw/NWO; grant, 016.146.310). The work described in this study was carried out in the context of the Parelsnoer Institute (PSI). PSI is part of and funded by the Dutch Federation of University Medical Centers.
