# Supplementary material for: Prolonged Tpeak‐Tend interval is a risk factor for sudden cardiac death in adults with congenital heart disease
Source: Congenit Heart Dis. 2019 Oct 1;14(6):952–7. doi: 10.1111/chd.12847 (PMC7003836; doi:10.1111/chd.12847)
Supplement: Supplementary file 1 [file CHD-14-952-s001.pdf]

Supplemental material to:

Prolonged Tpeak-Tend Interval is a Risk Factor for Sudden Cardiac Death in Adults With Congenital Heart Disease

Jim T. Vehmeijer, MD[1]; Zeliha Koyak, MD, PhD[1]; A. Suzanne Vink MD[1], Werner Budts, MD, PhD[2]; Louise Harris, MBChB[3]; Candice K. Silversides, MD[3]; Erwin N. Oechslin, MD[3]; Aeilko H. Zwinderman PhD[4]; Barbara J.M. Mulder, MD, PhD[1, 5] and Joris R. de Groot, MD, PhD[1]

[1] Department of Clinical and Experimental Cardiology, Heart Center, Amsterdam University Medical Centers – University of Amsterdam, Amsterdam, the Netherlands

[2] Department of Cardiology, Universitair Ziekenhuis Leuven, Leuven, Belgium; Department of Cardiovascular Sciences, Katholieke Universiteit Leuven, Leuven, Belgium

[3] Division of Cardiology, Peter Munk Cardiac Centre, Toronto Congenital Cardiac Centre for Adults; University of Toronto, Toronto, Ontario, Canada

[4] Department of Clinical Epidemiology and Biostatistics, Amsterdam University Medical Centers – University of Amsterdam, The Netherlands

[5] Netherlands Heart Institute, Utrecht, the Netherlands

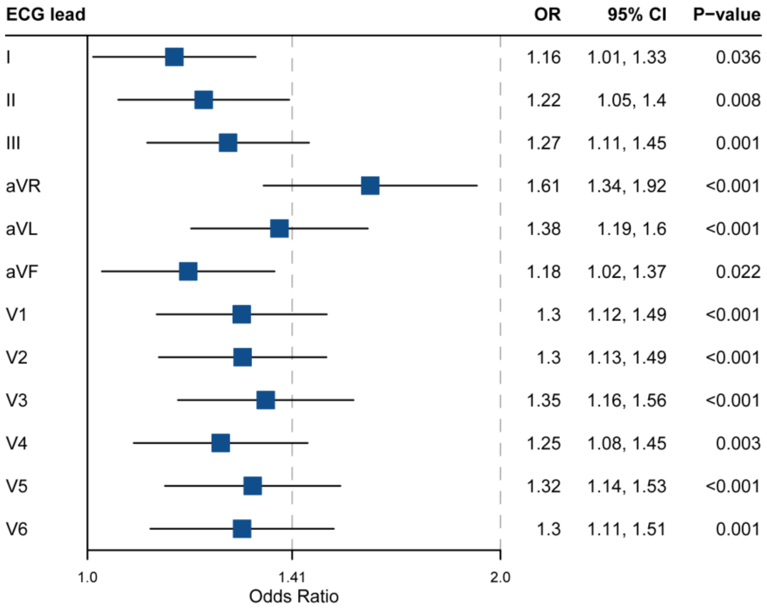

Supplemental figure S1: univariable odds ratios of sudden cardiac death per 10ms increase (<50ms to ≥90ms) of TpTe in different ECG leads

CI: confidence interval, OR: odds ratio

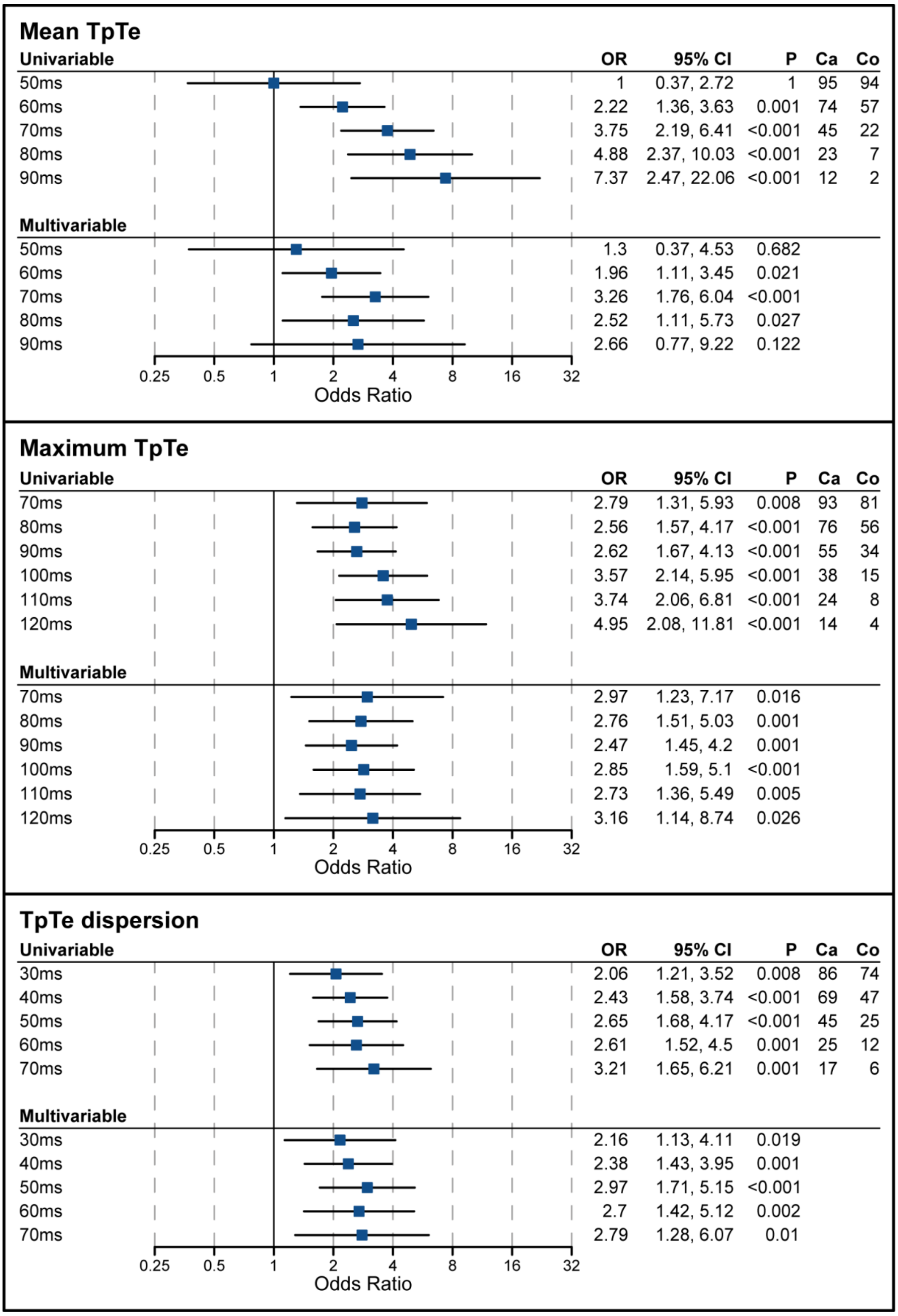

Supplemental figure S2: odds ratio for sudden cardiac death of several cutoff points of the mean TpTe, maximum TpTe and TpTe dispersion.

Multivariable: adjusted for impaired systemic ventricular function, heart failure symptoms and QRS-duration >120ms

Ca: percentage of cases above cutoff, CI: confidence interval, Co: percentage of controls above cutoff, OR: odds ratio

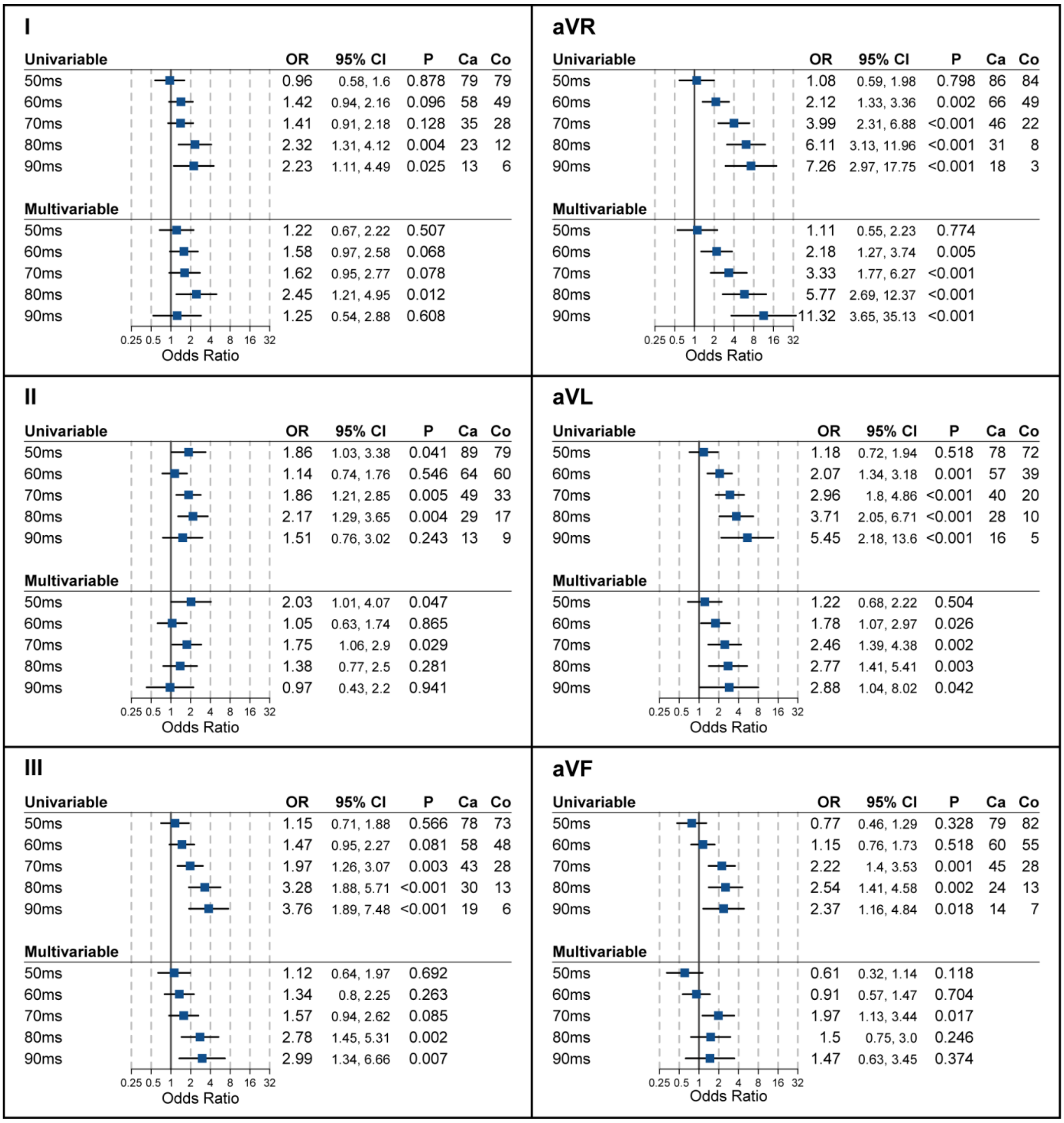

Supplemental figure S3a: odds ratios for SCD for different cutoffs of TpTe for leads I to aVF
Multivariable: adjusted for impaired systemic ventricular function, heart failure symptoms and QRS-duration >120ms
Ca: percentage of cases above cutoff, CI: confidence interval, Co: percentage of controls above cutoff, OR: odds ratio

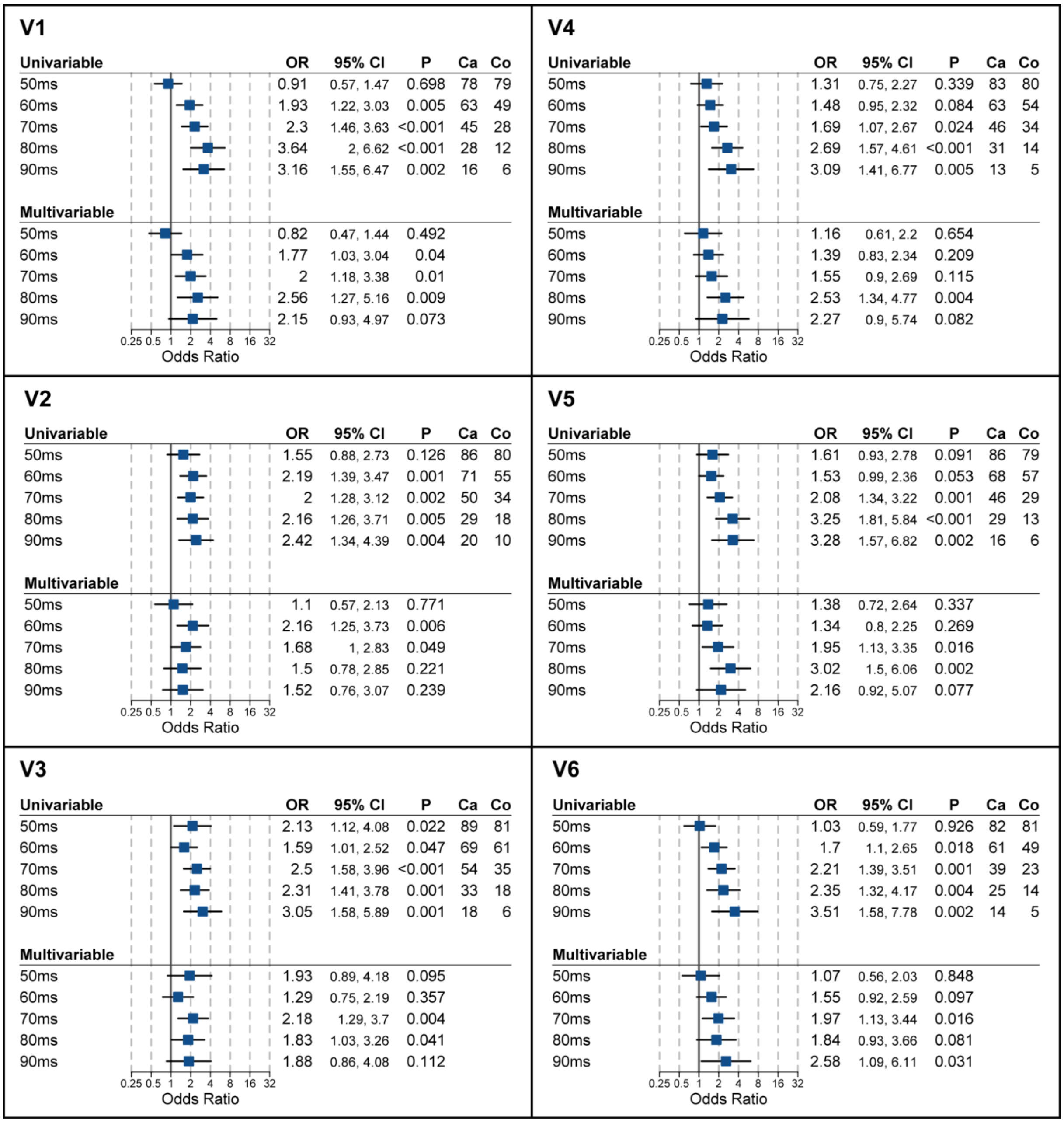

Supplemental figure S3b: odds ratios for SCD for different cutoffs of TpTe for leads V1 to V6
Multivariable: adjusted for impaired systemic ventricular function, heart failure symptoms and QRS-duration >120ms
Ca: percentage of cases above cutoff, CI: confidence interval, Co: percentage of controls above cutoff, OR: odds ratio
